# Extracellular signal‐regulated kinase 2 has duality in function between neuronal and astrocyte expression following neonatal hypoxic–ischaemic cerebral injury

**DOI:** 10.1113/JP275649

**Published:** 2018-07-11

**Authors:** Laura Thei, Eridan Rocha‐Ferreira, Donald Peebles, Gennadij Raivich, Mariya Hristova

**Affiliations:** ^1^ UCL Institute for Women's Health Maternal and Fetal Medicine Perinatal Brain Repair Group London WC1E 6HX UK; ^2^ School of Pharmacy University of Reading Reading RG6 6UA UK; ^3^ Institute of Clinical Sciences University of Gothenburg Gothenburg SE 416 85 Sweden

**Keywords:** Neonate, Hypoxia, ischemia, Brain, extracellular signal‐related kinase, Neuron, Astrocyte

## Abstract

**Key points:**

- This study identifies phosphorylated extracellular signal‐regulated kinase (ERK) to be immediately diminished followed by a rapid if transient increase for up to 4 h following hypoxic–ischaemic insult (HI) in the neonatal mouse.
- Phosphorylated ERK up‐regulation was prevented with systemic injection of the mitogen‐activated protein kinase kinase (MEK) inhibitor SL327. Treatment with SL327 both pre‐ and post‐HI gave a strong reduction in the number of dying cells and microgliosis.
- By utilising transgenic mouse mutations, we observe that neuronal ERK2 significantly contributes to tissue damage, while ERK1 and astrocytic ERK2 are neuroprotective.
- Compared to global inactivation, selective cell‐specific interference with ERK activity could result in stronger neuroprotection.

**Abstract:**

Hypoxia–ischaemia (HI) is a major cause of neonatal brain injury resulting in cerebral palsy, epilepsy, cognitive impairment and other neurological disabilities. The role of extracellular signal‐regulated kinase (ERK) isoforms and their mitogen‐activated protein kinase kinase (MEK)‐dependent phosphorylation in HI has previously been explored but remains unresolved at cellular level. This is pertinent given the growing awareness of the role of non‐neuronal cells in neuroprotection. Using a modified Rice–Vannucci model of HI in the neonatal mouse we observed time‐ and cell‐dependent ERK phosphorylation (pERK), with strongly up‐regulated pERK immunoreactivity first in periventricular white matter axons within 15–45 min of HI, followed by forebrain astrocytes and neurons (1–4 h post‐HI), and return to baseline by 16 h. We explored the effects of pharmacological ERK blockade through the MEK inhibitor SL327 on neonatal HI‐brain damage following HI alone (30 or 60 min) or lipopolysaccharide (LPS)‐sensitised HI insult (30 min). Global inhibition of ERK phosphorylation with systemically applied SL327 abolished forebrain pERK immunoreactivity, and significantly reduced cell death and associated microglial activation at 48 h post‐HI. We then explored the effects of cell‐specific ERK2 deletion alone or in combination with global ERK1 knockout under the same conditions of HI insult. Neuronal ERK2 deletion strongly decreased infarct size, neuronal cell death and microglial activation in grey matter following both HI alone or LPS‐sensitised HI. ERK1 deletion attenuated the protective effect of neuronal ERK2 deletion. Removal of astroglial ERK2 produced a reverse response, with a 3‐ to 4‐fold increase in microglial activation and cell death. Our data suggest a cell‐specific and time‐dependent role of ERK in neonatal HI, with a predominant, neurotoxic effect of neuronal ERK2, which is counteracted by neuroprotection by ERK1 and astrocytic ERK2. Overall, global pharmacological inhibition of ERK phosphorylation is strongly neuroprotective.

## Key points

- This study identifies phosphorylated extracellular signal‐regulated kinase (ERK) to be immediately diminished followed by a rapid if transient increase for up to 4 h following hypoxic–ischaemic insult (HI) in the neonatal mouse.
- Phosphorylated ERK up‐regulation was prevented with systemic injection of the mitogen‐activated protein kinase kinase (MEK) inhibitor SL327. Treatment with SL327 both pre‐ and post‐HI gave a strong reduction in the number of dying cells and microgliosis.
- By utilising transgenic mouse mutations, we observe that neuronal ERK2 significantly contributes to tissue damage, while ERK1 and astrocytic ERK2 are neuroprotective.
- Compared to global inactivation, selective cell‐specific interference with ERK activity could result in stronger neuroprotection.

## Introduction

Cerebral hypoxia–ischaemia (HI) is a leading cause of neurological deficits in neonates. It affects 1–5 per 1000 live births worldwide (Vannucci, 1990; Vannucci & Hagberg, 2004). The pattern of damage depends on the severity, gestational age, as well as antenatal/perinatal factors such as maternal/fetal infection (Vincer *et al*. 2006; Higgins & Shankaran, 2009).

The immature brain is particularly vulnerable to HI due to insufficient anti‐oxidant and scavenging systems preventing the elimination of endogenous free radicals (Ferriero *et al*. 1996; Volpe, 2001; Vannucci & Hagberg, 2004). The subcortical white matter of the immature brain is unmyelinated and predominantly populated with oligodendrocyte (ODC) precursors susceptible to an imbalance between pro‐ and anti‐inflammatory cytokines (Skoff *et al*. 2001; Dewar *et al*. 2003; Bain *et al*. 2010). Alteration in that cytokine balance shifts the differentiation of ODC precursors towards astrocytes instead of oligodendrocytes, thus affecting subsequent myelination (Bain *et al*. 2010). Additionally, inflammatory changes in the white matter also activate resident microglia causing release of neurotoxic substances including nitric oxide (NO), reactive oxygen species and pro‐inflammatory cytokines (Dommergues *et al*. 2003; Chock & Giffard, 2005; Polazzi & Monti, 2010; Kendall *et al*. 2011; Hagberg *et al*. 2012).

During neonatal HI multiple chemical stimuli, including growth factors, cytokines, glutamate and free radicals, interact with corresponding target cell membrane receptors (Irving & Bamford, 2002). Activation of their receptor‐linked tyrosine kinases stimulates signal transduction via the Ras/Raf/MEK1&2 pathway causing up‐regulation of phosphorylated extracellular signal‐regulated kinase 1 and 2 (pERK1&2), and affecting a range of transcription factors, protein kinases, cytoskeletal elements and regulators of apoptosis (Lu & Xu, 2006).

Previous studies of neonatal HI in rodents revealed phosphorylated ERK (pERK) positive neurons with signs of DNA damage at the core of infarct and the border zones to undamaged tissue (Wang *et al*. 2004). Pharmacological inhibitors of MEK/ERK (PD98059, U0126) reduced NO‐induced neuronal cell death following glutathione depletion *in vitro* (Canals *et al*. 2003; de Bernardo *et al*. 2004). In an adult mouse model of middle cerebral artery occlusion (MCAO), PD98059 administered prior to insult reduced infarct volume by 40–50%, accompanied by reduction in neurobehavioural defects (Alessandrini *et al*. 1999). A similar effect was observed in an adult gerbil model of cerebral ischaemia using the more selective MEK1/2 inhibitor UO126, with significant reduction in cerebral infarct and protection against hippocampal CA1 pyramidal neuron loss (Namura *et al*. 2001). On the other hand, administration of neuroprotective brain‐derived neurotrophic factor (BDNF) to postnatal day 7 (P7) HI mice resulted in a rapid increase in pERK and in phosphatidylinositol 3‐kinase (PI3K)/AKT. When ERK but not PI3K/AKT was inhibited, the neuroprotective effect of BDNF was abolished (Han & Holtzman, 2000).

Thus, despite the association of ERK activation to regions of neurodegeneration, the precise role of ERK1/2 in neonatal HI brain damage remains unclear. We hypothesise that ERK1 and ERK2 have cell‐ and time‐dependent effects following neonatal HI. The aim of our study was to explore the outcomes of cell‐specific ERK2 removal and global ERK1 deletion, as well as ERK1/2 inactivation in neonatal HI brain damage using a modified P7 Rice–Vannucci mouse model of HI insult.

## Methods

### Animals

**Figure 1:**
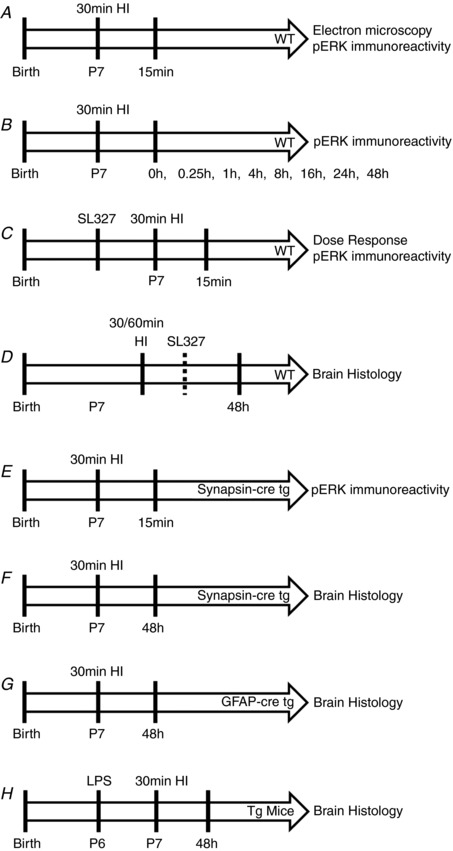
Schedule of experimental procedures *A*, WT (C57/Bl6) mice underwent 30 min hypoxic–ischaemic (HI) insult and were then killed at 15 min post‐hypoxia for pERK immunoreactivity evaluation. *B*, pERK immunoreactivity was assessed at multiple time points up to 48 h post‐insult to P7 WT mice. *C*, a dose response of SL327, controlled to vehicle alone, was administered 20 min prior to 30 min HI and pERK immunoreactivity was assessed at 15 min post‐insult. *D*, WT mice were subject to either 30 min or 60 min HI, with 133 μg/g SL327 or EtOH (vehicle) administered either 20 min prior to or 60 min post‐insult. Brain histology was assessed at 48 h. *E*, inhibition of neuronal pERK immunoreactivity was confirmed at 15 min post‐HI in synapsin‐cre driven ERK tg mutant mice compared to littermate WT controls. *F*, brain histology was assessed at 48 h after 30 min HI in synapsin‐cre driven ERK tg mutant mice and littermate WT controls. *G*, brain histology was assessed at 48 h after 30 min HI in GFAP‐cre driven ERK tg mutant mice and littermate WT controls. *H*, saline or LPS was injected at 12 h prior to 30 min HI in both synapsin‐cre and GFAP‐cre driven ERK tg mutant mice and littermate WT controls. Brain histology was assessed at 48 h.

All animal experiments and techniques were approved by the Ethics Committee of the University College London and were carried out by licensed personnel in accordance with the UK Home Office Guidelines (Animals (Scientific Procedures) Act, 1986). C57/Bl6 (Charles River, UK), ERK1^KO^, ERK2^ΔSyn^, ERK2^ΔGFAP^ and ERK1^KO^ERK2^ΔSyn^ mice were bred in‐house with a 12 h light/dark cycle and had free access to water and food. The ARRIVE guidelines (Kilkenny *et al*. 2010) were followed in all animal experiments. Animals were killed at different time points (see Fig. 1) post‐HI by intraperitoneal injection of sodium pentobarbital (2.5 μg/g) and confirmed by exsanguination.

Global ERK1 deletion was described by Nekrasova *et al*. (2005). Heterozygous ERK1 mice were bred together to produce both homozygous null mutants (ERK1^KO^) and wild‐type controls (ERK1^WT^). To ablate ERK2 expression in the CNS, and to overcome embryonic lethality of global ERK2 deletion, we utilised a cell‐specific approach with Cre recombinase driven transgenic mutation of LoxP site flanked ERK2 under the control of either the synapsin (ERK2^ΔSyn^) or glial fibrillary acidic protein (GFAP) (ERK2^ΔGFAP^) promoters in order to remove ERK2 expression from neurons or astrocytes, respectively. Syn‐Cre mice were provided by Dr Axel Behrens from the Mammalian Genetics Laboratory, Cancer Research UK, and animals expressing Cre recombinase under the control of GFAP promoter (GFAP‐Cre) were from Jackson Labs (USA, http://jaxmice.jax.org/strain/004600.html). Mice were bred heterozygous with C57/Bl6 to produce both homozygous null mutants (ERK2^ΔSyn^ or ERK2*^ΔGFAP^*) and wild‐type littermate controls (ERK2^WT^). Deletion of both ERK1 and 2 in neurons or astrocytes was achieved by breeding heterozygous ERK1 mutants with ERK2*^ΔSyn^* or ERK2*^ΔGFAP^*, respectively.

### DNA extraction and genotyping

DNA extraction was performed with the ‘Wizard’ Genomic DNA purification system according to the manufacturer's instructions (Promega, Southampton, UK), using mouse tail tips taken before the perfusion. Specific oligonucleotide primers (Invitrogen, Loughborough, UK) were used for genotyping against erk1, erk2, synapsin‐cre, GFAP‐cre.

### Hypoxia–ischaemia surgery

Surgeries were performed on C57/Bl6 and transgenic ERK mutant mice at P7, the age equivalent of late preterm human brain maturation. Animals were anaesthetised with isoflurane (5% induction, 1.5% maintenance), the left common carotid artery permanently occluded and the wound closed with tissue glue. The mice were returned to the dam for 2 h of recovery before being placed in a hypoxia chamber and exposed to continuous humidified 8% oxygen–92% nitrogen at 36°C for 30 or 60 min. The mice were left to recover at 36°C and returned to the dam for 2 h until being killed at 48 h post‐HI for analysis of neuropathological markers.

We have previously shown that lipopolysaccharide (LPS) bacterial endotoxin administered 12 h before the start of HI surgery confers a sensitising effect, as seen through a significant increase in neuropathological markers, when compared to saline‐injected controls (Rocha‐Ferreira *et al*. 2015). This is in concordance with other studies that show this sensitisation is mediated by the binding of LPS to toll‐like receptor 4 (TLR4) and recruitment of MyD88 adaptor protein (Wang *et al*. 2009). In the current study, P6 mouse pups were injected intraperitoneally (i.p.) with a single dose of LPS (serotype 055:B5, Fluka, Loughborough, UK; 0.6 μg/g), or saline as control (10 μl/g body weight (BW)). Twelve hours following injection, animals were exposed to carotid occlusion and 30 min hypoxia as above.

All animals were operated blindly with genotypes established after killing.

See Fig. 1 for all experimental outlines.

### Pharmacological manipulation

#### SL327 toxicity

SL327 (Tocris, Bristol, UK), a MEK1/2 inhibitor (Atkins *et al*. 1998) proved to be toxic when dissolved in DMSO (data not shown), and therefore, was dissolved in 100% EtOH instead. P7 C57/Bl6 mice were treated with a single i.p. injection of SL327 (Dommergues *et al*. 2003) at 0, 15, 30, 65 or 133 μg/g BW (*n* = 5 per group) 20 min prior to the start of 30 min hypoxia. Fifteen minutes after HI, brains were assessed for intensity of pERK staining.

#### Time window for pharmacological ERK inhibition

In order to investigate the effect of MEK1/2 inhibition on the brain regions of interest – isocortex, pyriform cortex, hippocampus, striatum, thalamus and external capsule – P7 C57/Bl6 animals were injected with a single i.p. dose of SL327 (133 μg/g BW) either 20 min preceding or 1 h following both 30 and 60 min hypoxia (*n* = 10 per group). Control animals received the corresponding volume of 100% EtOH alone (10 μl/g BW). Following 48 h survival, animals were killed and brains collected for histopathological analysis of tissue infarction (Nissl), cell fragmentation (TUNEL), microglial activation (CD11b) and astrogliosis (GFAP).

### Tissue sample preparation

For immunohistochemistry (IHC) analysis all animals were perfused with 4% paraformaldehyde (PFA)/PBS. The brains were excised, post‐fixed in 4% PFA/PBS for 1 h at 4°C, followed by cryoprotection for 24 h in 30% sucrose/PBS at 4°C and snap frozen on dry ice. Brains were sectioned on a cryostat into 40 μm sequential coronal sections starting from the point of fusion of the corpus callosum, in a total of 50 serial sections terminating in the hippocampus.

For electron microscopy, tissue was cut and stained as previously described (Hristova *et al*. 2010). In brief: glutaraldehyde‐fixed forebrain vibrotome sections of 100 μm were rinsed overnight in 2% sodium acetate solution and then pre‐treated for 6 h in 10% thioglycolic acid. Sections were developed for 25–30 min under visual control in a physical developer suspension containing 0.1% AgNO_3_, 0.1% NH_4_NO_3_, 0.5% H_4_SiW_12_O_40_ (silicotungstic acid) and 0.9% paraformaldehyde in distilled water, to which concentrated, aqueous Na_2_CO_3_ solution was added under vigorous stirring, to a final concentration of 2.5% Na_2_CO_3_. Following 2 min fixation and wash with 1% acetic acid, silver deposits were replaced with gold by immersing sections in 0.02% AuCl_3_ for 10 min. Sections were washed again for 10 min in two changes of 1% acetic acid, and 10 min in two changes of 2% sodium acetate, before transfer back into glutaraldehyde fixative until further use.

### Immunohistochemistry: frozen

Tissue IHC staining was performed as previously described (Hristova *et al*. 2010). In brief, sections were fixed in 4% formaldehyde/0.1 M phosphate buffer for 5 min before acetone treatment for antigen retrieval (50, 100, 50%: 2 min each). Sections were incubated with CD11b (αMβ2) (1:5000, Serotec, Kidlington, UK), glial fibrillary acidic protein (GFAP, 1:6000, Dako, Santa Clara, CA, US), pERK (1:100, Cell Signalling Systems, Hitchen, UK) and pC‐Jun (1:200, Santa Cruz Biotechnology, Heidleberg, UK) primary antibodies overnight at 4°C. Sections were then incubated with biotinylated secondary antibody (1:100 anti‐rabbit or anti‐rat IgG, Vector Laboratories, Inc., Burlingame, CA, USA) and visualised with avidin‐biotinylated peroxidase complex (ABC, Vector Laboratories, Peterborough, UK) and diaminobenzidine/H_2_O_2_ stain. Lastly, sections were air‐dried, immersed in xylene and covered using DEPEX (Fluka).

### Immunohistochemistry: free floating

This technique was utilised to stain for phosphorylated ERK due to the polyclonal primary antibody's sensitivity to dehydration. Sections were washed in PBS and endogenous peroxidases were blocked using 3% H_2_O_2_ in dH_2_O for 10 min at room temperature (RT). Antigen retrieval was achieved using 0.3% Triton (Triton X‐100, Bio‐Rad, Kidlington, UK) in a 5% goat serum/PBS solution for 30 min. Sections were incubated in wells containing polyclonal pERK antibody (1:100 in 5% goat serum/PBS) overnight at 4°C. Samples were washed in PBS and incubated for 1 h with secondary goat‐anti‐rabbit antibody (1:6000 dilution), before ABC incubation for 1 h at RT. The stain was visualised with 3,3'‐diaminobenzidine (DAB)/H_2_O_2_ and the reaction was stopped in dH_2_O and covered as described above.

### Electron microscopy silver–gold intensification

Brain sections were osmicated in reduced osmium tetroxide, dehydrated in serial dilutions of ethanol and embedded in Epon 208 (Taab Laboratories, Aldermaston, UK). Semi‐thin sections were counterstained with toluidine blue for light microscopy, and ultrathin, 80 nm sections were counterstained with uranyl acetate and lead citrate for examination in a JEOL 1010 transmission electron microscope.

### Histological analysis

Forty‐eight hours after HI insult, a time point where secondary energy failure has already initiated (Inder & Volpe, 2000), and with the highest level of widespread neuronal caspase‐3 expression within the acute period of injury (Johnston *et al*. 2011), the differences in microglial activation, astrocyte recruitment, infarct size and cell death were compared between littermate control animals and the corresponding SL327 treated, or mutant animals. All tissue sections were analysed blindly.

#### CD11b score

Activated microglia scores were assigned as previously described (Kendall *et al*. 2006; Hristova *et al*. 2016). In brief, a grade between 0 and 4 was assigned to each section for αMβ2 (0 = no activation, 1 = foci of non‐ramified active microglia, 2 = <50% coverage of active microglia, 3 = widespread active and predominantly phagocytic microglia, 4 = near full coverage of active and predominantly phagocytic microglia in addition to tissue infarct).

#### TUNEL+ cell death

Brain sections were stained at 400 μm intervals for DNA fragmentation using a TUNEL kit (Roche, Welwyn Garden City, UK) according to the manufacturer's instructions. Cell death was quantified at ×20 magnification by counting the number of TUNEL positive nuclei per brain region (3 microscope fields/region) of experimental and control animals.

#### Infarct volume measurement

Infarct volume was measured in 10 coronal sections at 400 μm intervals, stained with cresyl violet (Nissl stain). Optimas 6.2 image analysis software (Meyer Instruments Inc.) was utilised to calculate the intact brain tissue of each forebrain region by converting the measured injured and uninjured areas into square millimetres and then converting to a volume by multiplying by 400 μm. The sum of these volumes was converted into a percentage of surviving brain tissue by: injured/uninjured volume × 100 (Kendall *et al*. 2006).

#### Optical luminosity

A Sony AVT‐Horn camera was used to capture three 8‐bit RBG images of each of our regions of interest under a ×20 magnification with three eye fields/region. Images were then imported into the Optimas v6.5 Software. The mean and standard deviation (SD) of luminosity was obtained through the regions (Carsten Möller *et al*. 1996). For each image, the SD was subtracted from the mean, and the resulting value further subtracted from the mean optical luminosity of the empty glass slide. This provides a specific optical luminosity value (OLV).

### Statistics

When only two groups, experimental and control, were present, statistical analysis for tissue loss, cell death, CD11b and GFAP immunoreactivity in HI forebrain regions was performed by either a two‐tailed, unpaired Student's *t* test or two‐way ANOVA, *post hoc* Sidak. When more than two groups were present, statistical analysis was performed using one‐way ANOVA followed by *post hoc* Tukey. The confidence interval for all assessments was set at 95%. For a global hemispheric response, statistical significance was assessed through one‐way ANOVA, *post hoc* Tukey. Animals of different genotypes or treatment were compared in their response to injury using the combined regions ipsilateral to carotid artery occlusion.

## Results

### Rapid ERK phosphorylation occurs across whole brain in the neonatal mouse brain following HI

**Figure 2:**
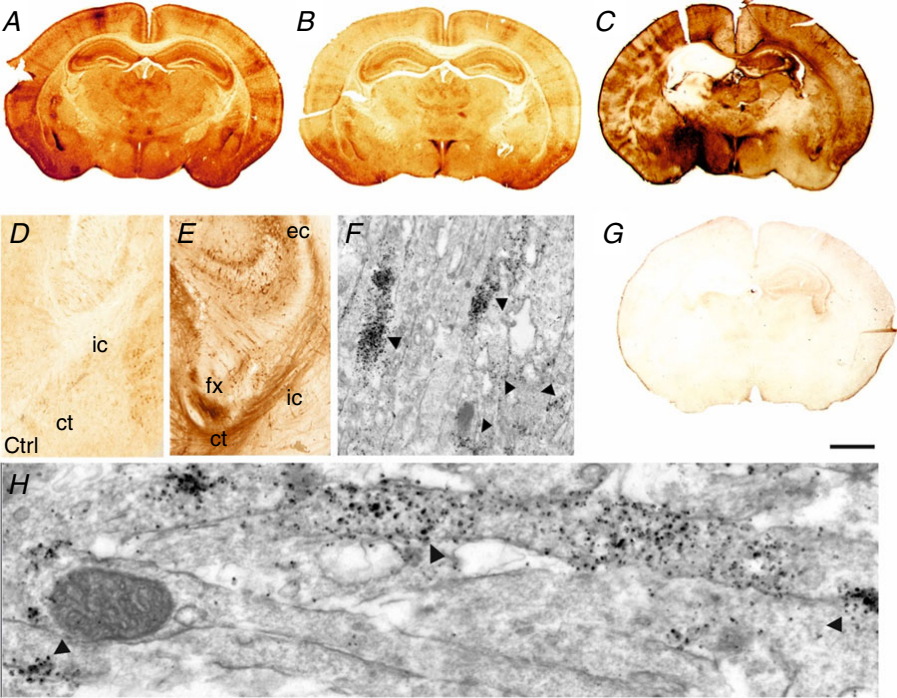
Histological and electron microscopy assesment of pERK expression in P7 mouse forebrain following 30 min HI *A* and *B*, distribution of normal pERK immunoreactivity in the forebrain of sham animal (*A*) and an animal with unilateral carotid occlusion (*B*). *C* and *G*, increased pERK immunoreactivity in untreated animals at 15 min following 30 min HI (*C*). Response was ablated with the application of MEK inhibitor SL327 (133 μg/g BW) (*G*). *D* and *E*, schematic summary of white matter pERK‐IR in naive (*D*) and after a 30 min HI insult (*E*). Light microscopy overview at the intersection between hippocampus (top), thalamus (left) and cerebral cortex (right), coronal section at mid‐parietal level (*D* and *E*). Note the faint pERK‐IR in control animal with carotid occlusion only (*D*, Ctrl), and the strong increase of expression in fibre tracts in external capsule (ec), fornix (fx), cortico‐thalamic fibres (ct), and descending tracts of the internal capsule (ic) at 1 h recovery following HI (*E*). *F* and *H*, electron microscopy of the internal capsule, at 15 min recovery following HI. Early pERK reactivity is located to the axons only. Arrows point to pERK positive clusters within adjacent axons. Scale bar (*A–C, G*): 1.5 mm [Color figure can be viewed at http://wileyonlinelibrary.com]

ERK activation in response to HI was visualised using immunohistochemistry for pERK immunoreactivity (pERK‐IR) in C57/Bl6 mouse pups at postnatal day 7 (P7). These animals underwent unilateral left carotid artery occlusion (CROC) and were exposed to 8% O_2_ for 30 min. The distribution of normal pERK‐IR in the forebrain of a sham‐operated animal is shown in Fig. 2 *A*. OLV for pERK immunoreactivity in animals with CROC (ischaemic insult) only (Fig. 2 *B*) was unchanged compared to sham operated littermates and were referred to as controls (or CTRL in Fig. 2 *D*). Intensity of pERK‐IR was comparable in both ipsilateral (occluded, hypoxic–ischaemic insult) and contralateral (non‐occluded, ischaemia alone) hemispheres.

Compared to controls, the animals exposed to both carotid occlusion and 30 min hypoxia exhibited a rapid increase in white matter pERK‐IR, reaching a maximum at 15 min (Fig. 2 *C*). Up to a 2‐fold increase in pERK‐IR was observed in cortical and subcortical white matter tracts (Fig. 2 *D* and *E*).

### White matter pERK expression is isolated to clusters within parallel axonal tracts

**Figure 3:**
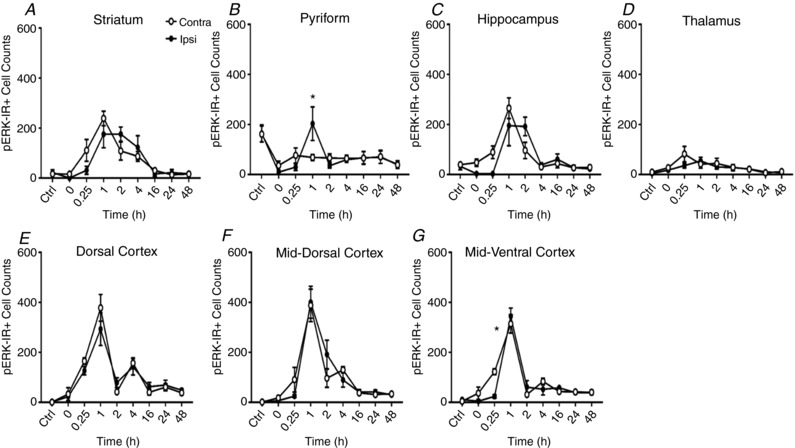
Number of phosphorylated ERK‐positive cells following HI insult per ×20 microscopy field (mean + SEM, *n* = 4 animals per time point) in forebrain regions Striatum (*A*), pyriform (*B*), hippocampus (*C*), thalamus (*D*), dorsal cerebral cortex (*E*), mid‐dorsal cortex (*F*) and mid‐ventral cerebral cortex (*G*). HI insult induces drastic changes in ERK phosphorylation in postnatal mouse forebrain, causing an initial ipsilateral blanking out (0–15 min) and then a bilateral peak at 1–2 h. ^*^ *P* < 0.05 in paired Student's *t* test for ipsilateral *versus* contralateral hemispheres.

To determine the precise ultrastructural pERK localisation within the subcortical white matter, pERK immunoreactivity augmented with silver–gold intensification (Hristova *et al*. 2010) revealed pERK clusters 200–500 nm in size, occurring in 1–2 μm‐long segments within central axons (Fig. 2 *F*). The segments of pERK expression within axons run parallel with neighbouring axonal segments within the white matter, with pERK clusters sometimes also in two adjacent axons (Fig. 2 *H*, black arrows). This fibre tract labelling disappeared within 60 min post‐HI, but was contrasted by a cell body pERK‐IR+ labelling in grey matter forebrain areas with a more delayed response (Fig. 3).

### Grey matter pERK is bilaterally increased following HI, with a common temporal pattern and peak expression at 1 h post‐insult

With the exception of thalamus, there is a strong increase in pERK‐IR with a peak at 1 h following recovery from HI, declining back to approximately baseline levels at 16 h (Fig. 3 *A–G*). A degree of regional specificity was observed with reference to pERK+ cells. While the striatum, pyriform cortex, hippocampus, dorsal, mid‐dorsal and mid‐ventral cortex all displayed a robust increase in pERK+ cells; the thalamus did not (Fig. 3 *D*). Of interest, the brain hemisphere contralateral to CROC saw a mildly, but not significantly, higher number of pERK+ cells compared to the ipsilateral. Sub‐regional exceptions were observed in pyriform cortex and mid‐ventral cortex where ipsilateral expression at 1 h was significantly higher (*P* = 0.00008 and *P* = 0.0001, respectively). Table 1 illustrates the peak number of pERK+ cells in grey matter regions of both hemispheres plus time of peak occurrence.

**Table 1:** pERK positive cell counts in grey matter regions of P7 mouse brains following 30 min HI

Region	Contralateral peak (mean ± SEM)	Peak time (h)	Ipsilateral peak (mean ± SEM)	Peak time (h)
Striatum	222.00 ± 30.53	1	202.86 ± 40.63	1
Pyriform	151.81 ± 25.77	0.25	304.38 ± 35.33	1
Hippocampus	269.69 ± 32.92	1	201.70 ± 34.33	2
Thalamus	63.93 ± 14.46	0.25	53.40 ± 11.29	1
Dorsal cortex	376.59 ± 27.95	1	298.50 ± 40.81	1
Mid‐dorsal cortex	274.32 ± 40.57	1	403.73 ± 44.47	1
Mid‐ventral cortex	315.91 ± 35.49	1	345.83 ± 30.68	1

### MEK inhibitor SL327 is efficient in blocking ERK phosphorylation

**Figure 4:**
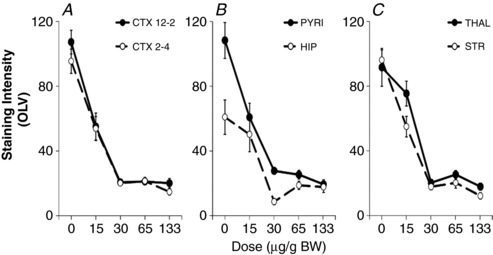
Ipsilateral ERK phosphorylation Dose response for SL327 inhibition of pERK immunoreactivity (optical luminosity value (OLV)), applied 20 min before a 30 min HI insult in hemispheric regions ipsilateral to carotid occlusion. *A*, CTX 12–2 dorsal cerebral cortex (12 to 2 o'clock segment), CTX 2–4 middle cerebral cortex (2 to 4 o'clock segment). *B*, PYRI: pyriform cortex, HIP: hippocampus. *C*, THAL: thalamus, STR; striatum. Increasing the dose of SL327 from 15 to 30 μg/g BW correlates to an 80% reduction in immunoreactivity.

Previous studies of ERK inhibition in rat neonatal HI injury introduced the MEK‐selective inhibitor UO126 intracerebroventricularly (i.c.v.) in two doses prior to CROC and 2.5 h hypoxia (Han & Holtzman, 2000). Due to the potentially traumatic nature of i.c.v. administration, we wished to examine the efficacy of our inhibitor, SL327, an analogue of UO126, when introduced via the intraperitoneal route. To determine the optimal dose and subsequent dose–response curve for pERK inhibition, five groups of animals (*n* = 5/group) were pre‐exposed to a step‐wise, 2‐fold dilution of 133 μg/g (i.e. 15, 30, 65, 133 μg/g) or to 100% EtOH (vehicle) alone (dose 0). As shown in Fig. 4 *A–C*, increasing doses of SL327 led to reduced pERK‐IR across six forebrain regions, with reliable suppression of immunoreactivity at 133 μg/g.

### Pre‐treatment with SL327 significantly reduces damage after HI insult

**Figure 5:**
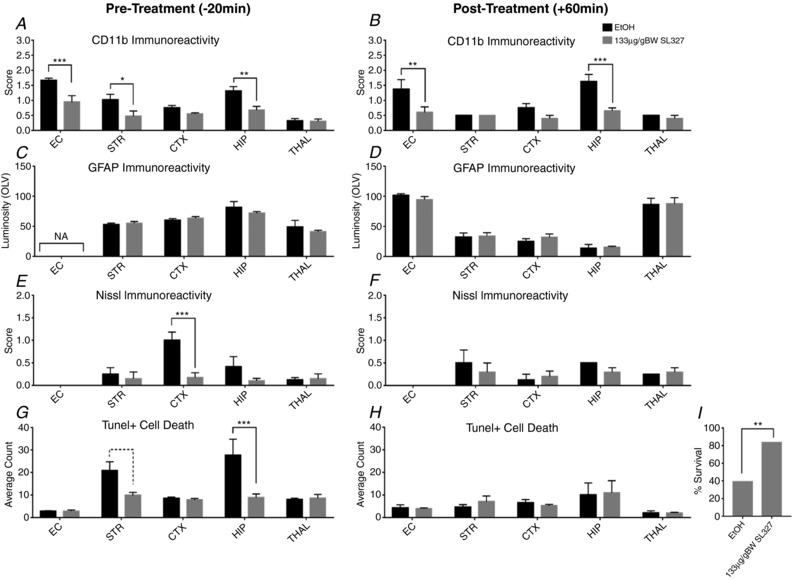
Effect of SL327 on αMβ2+ microglial activation, astroglial activation, neuronal tissue loss (Nissl body presence) and TUNEL+ cell death, when applied 20 min before (*A*, *C*, *E*, *G*) or 1 h post (*B*, *D*, *F*, *H*, *I*) 30 or 60 min HI Assessment at ×20 microscopy field magnification (mean + SEM over 3 fields). *A* and *B*, the levels of CD11b+ microglia are significantly decreased in the SL327 group in white matter (EC) as well as in most grey matter regions (STR, CTX, HIP). *E* and *F*, Nissl score was decreased in pre‐treated animals. Cortex was particularly spared compared to vehicle alone. *G* and *H*, this trend to decrease is observed with number of TUNEL+ cells. SL327 treated pups have a reduction in dying cells compared to EtOH treated animals, significantly so in pre‐treated STR and HIP. *C* and *D*, extent of gliosis and reactive (GFAP+) astrocyte activation was unaffected by the application of SL327. ^*^ *P* < 0.05, ^**^ *P* < 0.01, and ^***^ *P* < 0.001 using ANOVA and *post hoc* Tukey. *I*, χ^2^ analysis showed survival rate was significantly increased in animals treated with SL327 1 h following a more severe 60 min HI.

To determine the effect of pERK inhibition on neonatal HI brain damage, P7 mouse pups (C57/Bl6) were pre‐exposed to SL327 (133 μg/g BW, *n* = 10) or EtOH (*n* = 8), 20 min prior to insult. Here, they were subject to a 30 min HI insult. Results were assessed 48 h post‐HI. Histopathological assessment, shown in Fig. 5, revealed that 20 min pre‐treatment with SL327 significantly reduced levels of microglial activation in all brain regions (apart from thalamus and cortex, Fig. 5 *A*), neuronal loss, via the presence of Nissl bodies in the cortex (*P* = 0.001, Fig. 5 *E*), and overall TUNEL+ cell death in striatum and hippocampus (Fig. 5 *G*, *P* = 0.06, *P* = 0.0001). The levels of reactive astrogliosis assessed through OLV of GFAP‐IR remained unaffected (Fig. 5 *B*).

### Post‐treatment with SL327 reduces microglial activation but not HI damage

To examine the time dependent window for pharmacological pERK inhibition, a second set of P7 animals was injected 60 min post‐30 min HI with 133 μg/g BW SL327 or EtOH alone (*n* = 6 animals per group). Post‐treatment with SL327 caused a significant reduction of CD11b immunoreactivity in external capsule white matter (*P* = 0.01) and hippocampus (*P* = 0.0001, Fig. 5 *B*). Markers for either activated astrocytes, TUNEL+ cell death or neuronal loss remained unaffected (Fig. 5 *D*, *F* and *H*).

### A significant number of neonatal mice survived severe HI brain injury with SL327 treatment 1 h after insult

Previous work by our group and others have shown that a severe form of insult, exposure to 60 min hypoxia alone, leads to an almost complete loss of hippocampal neurons and severe tissue damage in cortex, thalamus and basal ganglia, as well as in the subcortical white matter (Lehnardt *et al*. 2003; Kendall *et al*. 2006). To test the efficacy of ERK inhibition in a severe HI insult to P7 mice following 60 min of hypoxia, 133 μg/g BW SL327 was injected 60 min after completing the hypoxia.

The control group (EtOH) showed a significantly higher incidence of death in the 16–48 h interval, with survival of only 9 of 23 animals at 48 h (39%, *P* = 0.002), compared to the SL327 treated group with a survival of 83% (Fig. 5 *I*). Despite this, cortex, pyriform cortex, hippocampus, striatum, thalamus and external capsule revealed little difference in microglial activation and TUNEL+ cell death in SL327‐treated pups when compared to vehicle‐treated controls when killed at 48 h (data not shown).

### Neuronal deletion of ERK2 has no effect on 30 min HI forebrain injury in the neonatal mouse

Due to embryonic lethality of the ERK2 null (−/−) phenotype (Yao *et al*. 2003; Satoh *et al*. 2007), the effect of ERK2 on neonatal HI brain injury was examined using cell type‐specific deletions with LoxP‐tagged (floxed) ERK2 genes and assessed alone and in combination with global ERK1 deletion. Mice with global ERK1 deletion (ERK1^KO^) were crossed with animals carrying the ERK2 gene flanked by LoxP sites on either side (ERK2^f/f^), and then further crossed with those expressing Cre recombinase under the control of neuronal synapsin promoter (Syn::Cre) (Ruff *et al*. 2012) or astroglial GFAP promoter (GFAP::Cre). Heterozygous breeding for Syn::Cre and ERK1^KO^ gave rise to four distinct genotypes used in the study: ERK2^f/f^ alone, which were functionally wild‐type (ERK1/2^WT^); Syn::Cre and ERK2^f/f^, where neurons lack ERK2 but ERK1 is expressed normally (ERK2^ΔSyn^); global deletion of ERK1 plus ERK2^f/f^ (ERK1^KO^); and Syn::Cre plus both ERK1^KO^ and ERK2^f/f^ (ERK1^KO^ERK2^ΔSyn^). All animals were genotyped after completing the HI experiment; heterozygous mice with the single wild‐type (WT) copy of ERK1 were excluded from assessment of 48 h histopathology.

**Figure 6:**
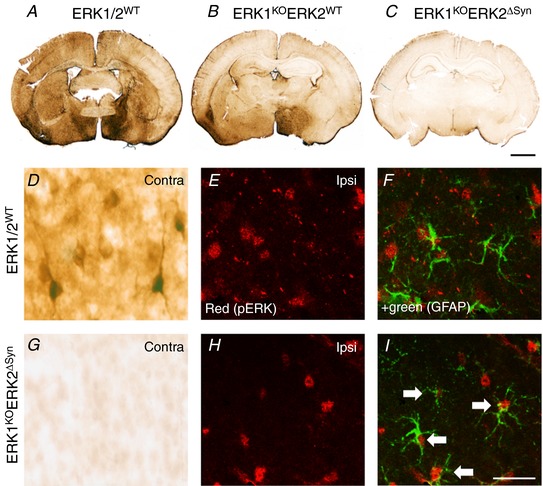
Effects of global ERK1 and neuronal ERK2 deletion on pERK immunoreactivity at 15 min post‐30 min HI insult *A*–*C*, control (ERK1/2^WT^) animal (*A*), global deletion of ERK1 and ERK2^WT^ (ERK1^KO^) (*B*), global ERK1 deletion and homozygous neuronal ERK2 deletion (ERK1^KO^ERK2^ΔSyn^) (*C*). *C*, pERK immunoreactivity is almost completely reduced following deletion of both copies of ERK1 and ERK2. *D–I*, quantification and distribution of pERK immunoreactivity at high magnification. *D*, pERK staining in the contralateral pyriform cortex of ERK1/2^WT^ with strong neuronal reactivity and prominent dendritic staining which disappears in the presence of global ERK1 deletion and homozygous neuronal ERK2 mutation ERK1^KO^ERK2^ΔSyn^. *E*–*I*, residual immunoreactivity on the ipsilateral side. *E* and *H*, pERK alone. *F* and *I*, immunofluorescence double labelling with GFAP demonstrating co‐localisation of pERK in astrocytes, particularly pronounced in ERK1^WT^ERK2^ΔSyn^ (white arrows). Scale bar = 25 µm [Color figure can be viewed at http://wileyonlinelibrary.com]

The effects of ERK2 deletion were first assessed through pERK‐IR at 15 min post 30 min HI insult, as shown in Fig. 6. Control animals showed similar pERK expression compared to WT as displayed in Figs 2 *A* and 6 *A*. Homozygous ERK1 deletion led to a visible decrease (Fig. 6 *B*), and deletion of both ERK2 copies in ERK1^KO^ERK2^ΔSyn^ to an almost complete disappearance of pERK‐IR (Fig. 6 *C*). At high resolution (Fig. 6 *D* and *G*) there was a complete disappearance of the neuronal pERK‐IR (Fig. 6 *G*) compared to ERK1/2^WT^ littermate controls (Fig. 6 *D*). In contrast, this homozygous deletion of ERK1 and neuron‐specific deletion of ERK2 did not interfere with the pERK+ astroglial cells (Fig. 6 *E–I*).

**Figure 7:**
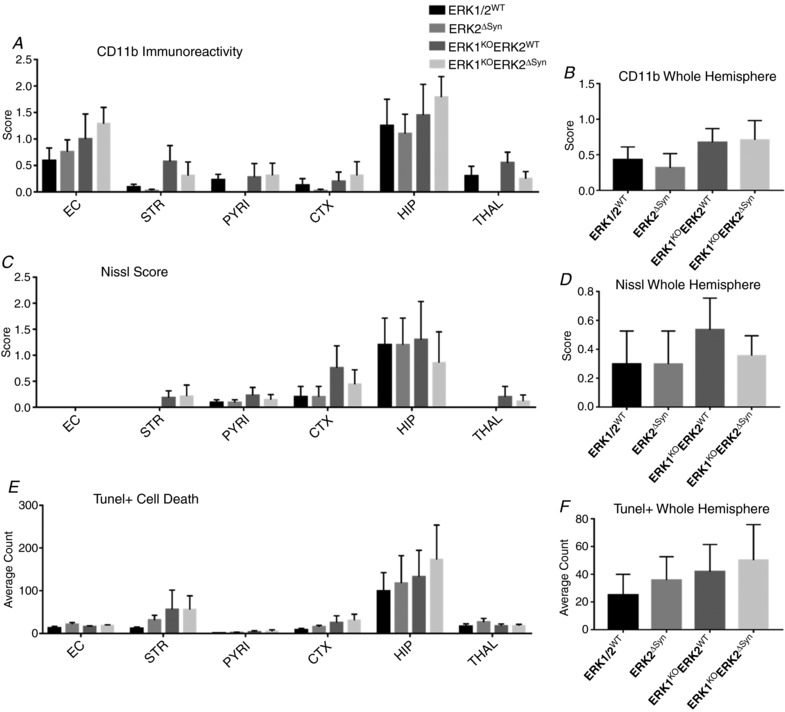
Forty‐eight hours post‐insult, damage markers were analysed in four neuronal ERK variations: ERK1/2^WT^, neuronal ERK2 deletion only (ERK1^WT^ERK2^ΔSyn^), global ERK1 deletion only (ERK1^KO^) and global ERK1 deletion plus neuronal ERK2 deletion (ERK1^KO^ERK2^ΔSyn^) Forebrain sections were analysed for microglial activation (*A* and *B*), Nissl score (*C* and *D*), and TUNEL+ cell death (*E* and *F*). *A*–*F*, for neuron‐specific transgenic mutants there was no significant change in damage markers for each sub‐region nor over the whole ipsilateral hemisphere. Despite this, inclusion of ERK1^KO^ gave a trend to increased injury over whole hemisphere (*B*, *D* and *F*). Analysis at ×20 eye field (mean + SEM over 3 fields) using ANOVA and *post hoc* Tukey.

In the mild HI model of 30 min hypoxia following carotid occlusion, homozygous global deletion of ERK1, alone or in combination with homozygous neuronal Syn::Cre‐mediated deletion of ERK2, did not lead to a significant change in histopathology, based on microglial (CD11b) activation score (Fig. 7 *A* and *B*), neuronal tissue loss (Fig. 7 *C* and *D*) and the number of TUNEL+ dying cells (Fig. 7 *E* and *F*). In total 24 pups at P7 were subject to 30 min HI with a survival time of 48 h, with the groups of ERK1/2^WT^ (*n* = 5 pups), ERK2^ΔSyn^ (*n* = 5), ERK1^KO^ (*n* = 6) and ERK1^KO^ERK2^ΔSyn^ (*n* = 8) at completion of the experiment. Both at a sub‐regional level, and over total hemisphere, changes in neuronal ERK expression gave no significant effect on damage markers.

### Astrocytic expression of ERK2 is required for neuroprotection following 30 min HI insult

To further investigate the role of astroglial ERK in HI, mice carrying the GFAP::Cre promoter were crossed with the ERK1^KO^ERK2^f/f^ (without Syn::Cre) for four generations to produce ERK1^KO^ERK2^ΔGFAP^ and ERK1^KO^ mouse pups. Post‐HI genotyping revealed 9 controls (ERK1/2^WT^) and 10 each for the ERK1^KO^ and ERK1^KO^ERK2^ΔGFAP^ groups.

**Figure 8:**
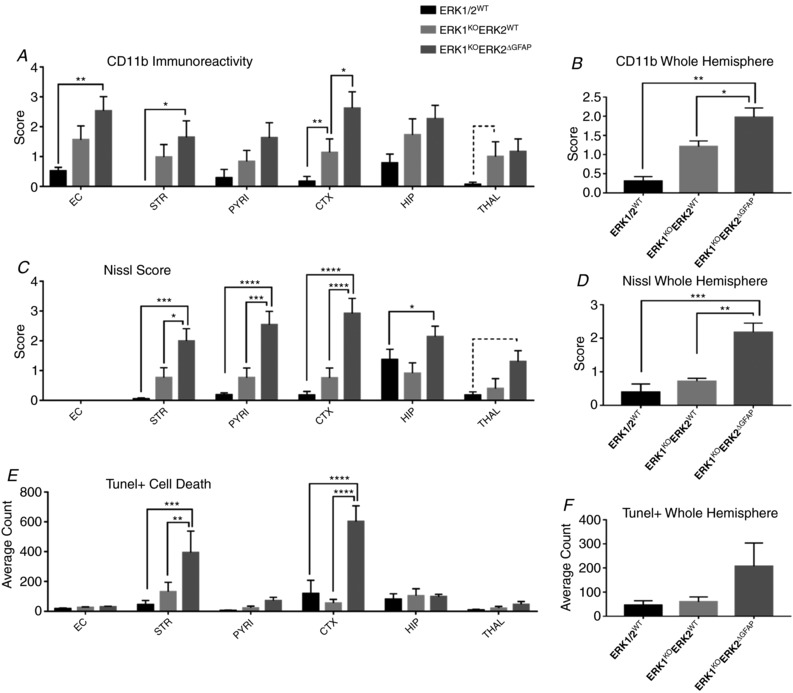
Three astrocytic mutant groups were assessed for brain damage markers: ERK1/2^WT^, ERK1^KO^ and ERK1^KO^ERK2^ΔGFAP^ Deletion of ERK1 resulted in a strong increase in microglial activation in the ipsilateral hemispheres of both ERK1^KO^ and ERK1^KO^ERK2^ΔGFAP^ groups compared to controls. *A* and *B*, increased ipsilateral microglia activation both in grey matter regions and over total hemisphere. Similarly, an increased level of Nissl bodies was observed (*C* and *D*), the exception being in thalamus where *P* = 0.06. *E* and *F*, increased density of TUNEL+ dying cells in striatum and cortex, although this is not reflected statistically in total hemisphere counts. Analysis at ×20 eye field (mean + SEM over 3 fields). ^*^ *P* < 0.05, ^**^ *P* < 0.01, ^***^ *P* < 0.001 and ^****^ *P* < 0.0001 using ANOVA and *post hoc* Tukey.

Global deletion of ERK1 alone was associated with higher averages for activated microglia in cortex (*P* = 0.01) and a trend to increase in thalamus (*P* = 0.06) compared to ERK1/2^WT^ littermate controls. However, the number of Nissl bodies and total cell death (TUNEL+) were not statistically changed (Fig. 8 *A*, *C* and *E*).

Selective deletion of both ERK2 gene copies in the GFAP‐expressing cells (ERK1^KO^ERK2^ΔGFAP^) resulted in a strong and robust increase in all damage markers, both on a regional basis and over total hemisphere (Fig. 8). All forebrain regions showed an increased presence of activated microglia, particularly in white matter where *P* = 0.01. More notably both tissue loss and cell death were greatly enhanced in grey matter regions (apart from thalamus), where striatum and cortex were most affected (Fig. 8 *C–F*). Nissl bodies were observed at a higher abundance in striatum (*P* = 0.001), pyriform cortex (*P* < 0.0001), cortex (*P* < 0.0001) and hippocampus (*P* = 0.04). TUNEL+ cell death was significantly increased in both striatum (*P* = 0.001) and cortex (*P* < 0.0001); however, this was reflected as a trend but with no statistical effect over the whole hemisphere (Fig. 8 *F*).

Interestingly, this effect was bilateral, with contralateral cortex of ERK1^KO^ERK2^ΔGFAP^ resulting in significant increase of microglial activation in regions contralateral to carotid occlusion with sub‐regional differences in external capsule (*P* = 0.03), pyriform cortex (*P* = 0.04), striatum (*P* = 0.03) and cortex (*P* = 0.01). In addition there was an increase in the overall number of TUNEL+ cells (*P* = 0.003) (data not shown).

### Neuronal ERK2 is a significant contributor to forebrain response after LPS‐sensitised HI insult in the P7 mouse

Compared to 30 min HI, systemic pre‐exposure to LPS endotoxin from *Escherichia coli* results in strongly increased tissue loss and neuronal and astroglial cell death (Kendall *et al*. 2011; Jã *et al*. 2013), mediated by the LPS/TLR4/MyD88 pathway induction of genes encoding pro‐inflammatory factors, particularly the tumour necrosis factor (TNF) family of cytokines (Wang *et al*. 2009; Kendall *et al*. 2011). In peripheral tissues, these effects involve ERK signalling (An *et al*. 2002; Watts *et al*. 2011). To determine the role of ERK1/2 in LPS‐sensitisation to cerebral HI insult, P6 mice were injected intraperitoneally with LPS (*E. coli* 055/B5 serotype) 0.6 μg/g BW 12 h prior to a 30 min insult.

**Figure 9:**
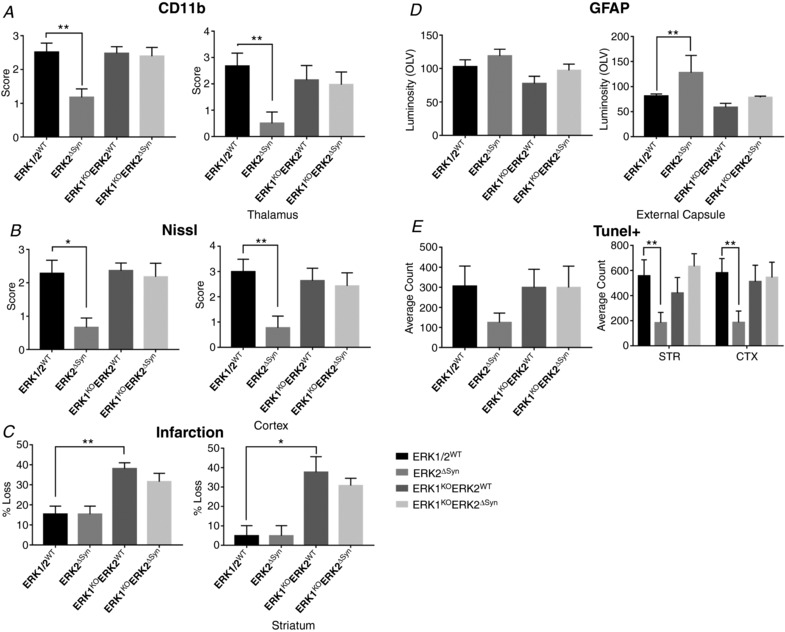
Neuronal ERK2 is required for non‐neuronal cell activation and recruitment after combined LPS and 30 min HI insult Neuronal ERK2 deletion, in the presence of WT ERK1 expression significantly reduces activated microglia (*A*), neuronal cell death (*B*), infarction (*C*) and astrogliosis (GFAP, *D*). In ERK2^ΔSyn^ mutants this response is significantly decreased. *E*, quantification of TUNEL+ dying cells. ERK2^ΔSyn^ exhibited a decrease in the number of dying cells; although not significant over the whole hemisphere, both striatum (STR) and cortex (CTX) saw a highly significant reduction in the number of TUNEL positive cells. Cell death patterns were not reflected in a decrease in lesion size compared to ERK1/2^WT^ controls. However, global deletion of ERK1 or double knockout animals saw a significant increase in lesion size and tissue loss with STR being most affected (*C*). Regions of significant changes are shown for each corresponding damage marker. Analysis at ×20 eye field (mean + SEM over 3 fields) using ANOVA and *post hoc* Tukey where ^*^ *P* < 0.05, ^**^ *P* < 0.01.

Recruitment of activated microglia over total hemisphere was strongly and significantly decreased in animals lacking neuronal ERK2 expression (*P* = 0.008, Fig. 9 *A*). ERK1 KO and double ERK KO were unaffected compared to littermate controls. Looking at individual regions, there is a significant decrease in thalamus (*P* = 0.01) with a similar trend, but not reaching significance, for pyriform cortex, hippocampus and striatum (not shown). Additional deletion of ERK1 (ERK1^KO^ERK2^ΔSyn^) completely abolished this ERK2^ΔSyn^ mediated reduction in microglial activation in total hemisphere (*P* = 0.048, Fig. 9 *A*).

Nissl and TUNEL+ cell death assessments (Fig. 9 *B* and *E*) in the ERK2^ΔSyn^ subgroup were similar to the ones observed in respect to microglial activation, i.e. reduction of both markers. Similarly to the trend observed in the microglial activation this reduction disappeared in ERK1^KO^ERK2^ΔSyn^. Using a one‐way ANOVA with *post hoc* TUKEY the number of Nissl bodies was significantly different in ERK2^ΔSyn^ animals only, with cortex (*P* = 0.02, Fig. 9 *B*) as the most affected region and an overall trend to decrease seen in all other grey matter regions (not shown). TUNEL+ cell counts were lower in all regions with single ERK2^ΔSyn^ mutations. Both striatum and cortex saw significant reduction (*P* = 0.009 and *P* = 0.008, respectively, Fig. 9 *E*), although counts over total hemisphere were not statistically significant. Interestingly this cumulatively did not contribute to total hemisphere tissue loss in the ERK2^ΔSyn^ cohort; what was observed, though, was a significant involvement of ERK1 mutations, either alone or in combination with neuronal ERK2 deletion, in infarction and overall loss (*P* = 0.009, Fig. 9 *C*) of 72%, particularly in striatum (*P* = 0.03, Fig. 9 *C*).

GFAP‐IR remains unaffected between all four mutant groups (Fig. 9 *D*), the exception being an increase in white matter GFAP expression (*P* = 0.001) in ERK2^ΔSyn^ animals. As before, this effect was ameliorated by the presence of ERK1^KO^ to baseline expression (Fig. 9 *D*).

### Astrocyte deletion of ERK1/2 is protective in cerebral cortex after LPS‐sensitised HI insult

We explored the effects of LPS‐sensitised 30 min HI insult and GFAP‐specific ERK2 deletion on ERK1 null background (ERK1^KO^ *n* = 6, ERK1^KO^ERK2^ΔGFAP^ *n* = 4) against WT littermate controls (*n* = 8).

**Figure 10:**
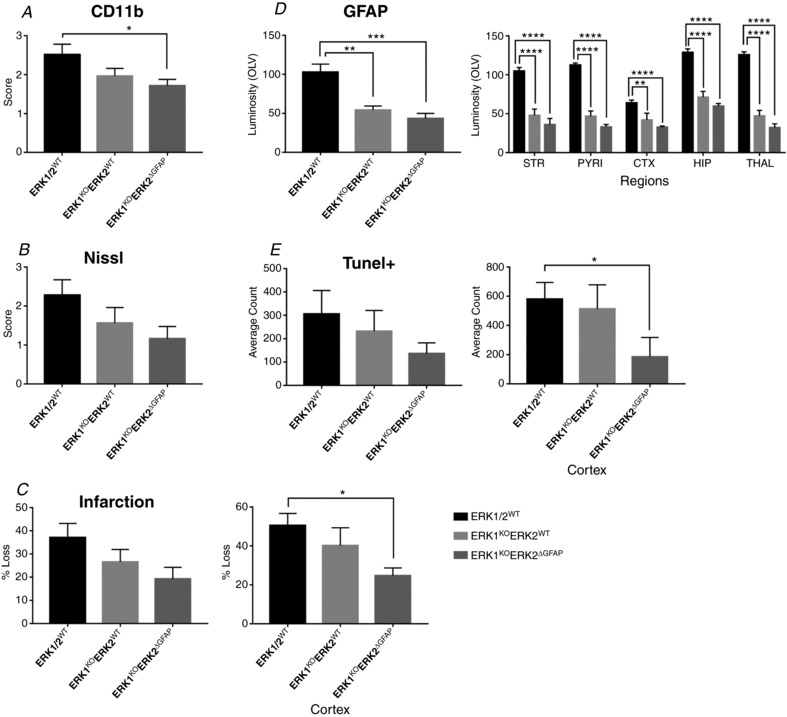
Astrocytic ERK directs astrogliosis and tissue loss after combined LPS and 30 min HI insult Global deletion of ERK1 had no direct effect on microglial activation (*A*), neuronal damage (*B*), tissue loss (*C*) or overall cell death (*E*). Double ERK mutations in astrocytes gave a reduction in microglial activation, cell death and tissue loss with cortex being the region most spared (*A*, *C* and *E*). ERK strongly drives astrogliosis in response to HI. Deletion of even one copy results in a dramatic decrease of GFAP, marker for gliosis, with significance seen in all grey matter regions (*D*). ^*^ *P* < 0.05, ^**^ *P* < 0.01, ^***^ *P* < 0.001, and ^****^ *P* < 0.0001 using ANOVA and *post hoc* Tukey.

CD11b‐IR was reduced in double mutant mice (*P* = 0.03), which was a cumulative effect with no individual region seeing significant change (Fig. 10 *A*). A similar trend was observed with Nissl bodies score (Fig. 10 *B*). TUNEL+ cell death was reduced in ERK1^KO^ERK2^ΔGFAP^ compared to WT littermate controls although significance was not reached. A strong contributor to this was a clear and significant sparing of the cortex (*P* = 0.03, Fig. 10 *E*) and this is confirmed by the same pattern of sparing observed by tissue loss, where damage to the cerebral cortex was prevented in the ERK1^KO^ERK2^ΔGFAP^ animals (*P* = 0.04, Fig. 10 *C*).

### ERK1/2 expression in the brain is a strong contributor to astrocyte activation and astrogliosis after LPS‐sensitised HI insult

In contrast to the mild effect on cell death, GFAP‐IR did show a strong and significant reduction (*P* < 0.01) in astrogliosis in all grey matter regions. This was true for both single ERK1 and double ERK1 and 2 mutant animals compared to littermate controls (Fig. 10 *D*). Whereas neuronal ERK2 deletion increases astrocyte reactivity in white matter (Fig. 9 *D*), astrocyte‐specific deletion of ERK2 has no effect on white matter (not shown). This is a robust indication that ERK phosphorylation is required for astrocyte activation and a master modulator of astrogliosis observed with neonatal brain injury.

## Discussion

During neonatal HI insult, once a critical threshold is reached, cellular mechanisms begin to fail due to ATP insufficiency leading to a two‐phase neurotoxic cascade (Sanders *et al*. 2010). Primary energy failure, resulting in immediate and necrotic cell death, is followed by a period of up to 6 h in which reperfusion can occur. Afterwards, secondary neuronal cell death ensues due to multiple molecular imbalances instigated by excitotoxic oxidative stress and mitochondria failure (Rocha‐Ferreira & Hristova, 2016). Depending on the severity of the insult, tertiary energy failure associated with late cell death, astrogliosis, as well as remodelling and repair, lasting for weeks and months following the initial HI insult may occur (Rocha‐Ferreira & Hristova, 2016). Owing to their extensive control over cellular physiology, the mitogen‐activated protein kinase kinases have a potential role in hypoxia induced cell death.

Our study shows rapid phosphorylation of ERK following mild HI insult. In line with previous studies, ipsilateral expression of pERK is immediately nullified for the first 15 min after insult followed by a rapid bilateral increase in expression that peaked at 1 h and returned to baseline by 4 h (Alessandrini *et al*. 1999; Wang *et al*. 2003, 2004; van den Tweel *et al*. 2006) establishing a consistent temporal pattern of pERK expression in neonatal rodent models of HI.

Initial pERK reduction was equally observed in both white and grey matter. The appearance of axonal pERK was seen from 15 min onwards, and normalised by 1 h (Fig. 2). Expression of pERK was observed in white matter, bound within the cytoplasm beyond 2 h post‐HI. Our data show clustering of pERK positive axons following HI, which has not been reported before (Fig. 2). This effect coincides with other *in vivo* studies where neurons showing cytoplasmic pERK activity are commonly adjacent to one another at the borders of ischaemia‐induced micro‐lesions in adult mice (Wang *et al*. 2003).

Pre‐treatment with SL327, a selective inhibitor of MEK, resulted in decreased levels of microglia activation, histological brain injury, and TUNEL+ cell death. These SL327 mediated protective outcomes were retained when administered up to 1 h post‐insult. This is in line with current protocols where pre‐treatment with U0126 to bilateral carotid artery occlusion reduced the loss of hippocampal neurons in addition to an overall decrease of infarct size associated with improved neurological outcome in adult rodents (Namura *et al*. 2001). Other *in vivo* studies explored the suppression of cytokine release following HI by application of U0126 both 20 min prior to and immediately following MCAO in the adult mouse (Namura *et al*. 2001). This suggests a mechanism by which the protective effects are due to restoration of the balance between pro‐ and anti‐inflammatory cytokines thus maintaining subsequent myelination (Bain *et al*. 2010).

Post‐treatment with SL327 after 60 min of 8% O_2_ exposure had diminished efficacy, compared to 30 min exposure, in protecting the neonatal mouse brain from ischaemic damage. Whilst markers for damage remained unaffected, the morbidity of this cohort was dramatically improved with survival rate increasing from 30% in EtOH treated controls to 83% in the SL327 group.

Some data suggest that transient suppression of ERK phosphorylation by an intraperitoneal injection of SL327 at P6 without a surgical procedure significantly increases cleaved caspase‐3 expression, and respectively apoptosis (Yufune *et al*. 2016). Nevertheless, our data are obtained using TUNEL+ cell death as a broader marker of damage (Hristova *et al*. 2010) having in mind that in neonatal HI apoptosis, necrosis and autophagy take place (Rocha‐Ferreira & Hristova, 2016). Thus SL327 might cause apoptosis, but at the same time prevent necrosis and autophagy thus having an overall neuroprotective function in neonatal HI. However, this requires further investigation.

Use of neuronal specific ERK2 knockout mice resulted in a clear and significant reduction of dying neurons, active microglia and brain injury with cortex, pyriform cortex and striatum being regions of particular sensitivity. The neuroprotection following ERK2 deletion correlates well with the actions of MEK inhibitors in adult MCAO studies (Alessandrini *et al*. 1999). Alessandrini's group showed that in the adult gerbil model of focal cerebral ischaemia, infarct size was reduced by 55%, suggesting that similarly to our results, ERK must be acting alongside other complementary or parallel pro‐apoptotic pathways in order to induce cell death after HI (Namura *et al*. 2001).

Global ERK1 deletion is embryonically viable with no phenotypic differences to wild‐type littermates. By deleting ERK1 globally, we observed an effect contrasting the neuroprotection achieved through neuronal ERK2 deletion, where ERK1 mutation increased HI brain damage following 30 min HI compared to littermate controls.

In adult mouse forebrain, expression of ERK1 is significantly lower than that of ERK2 – up to 6‐fold less in the frontal cortex (Ortiz *et al*. 1995). The complementary and ubiquitous co‐expression of ERK1 and 2 has led to the paradigm that ERK1 regulates ERK2 actions on cell growth and survival (Pouysségur & Lenormand, 2003; Lefloch *et al*. 2008). However, developmental complications have associated ERK1 with thrombocyte dysfunction (Nekrasova *et al*. 2005; Lefloch *et al*. 2008), as well as behavioural studies that link the MAP3K gene on chromosome 16, encoding ERK1, to altered synaptic plasticity and subsequent behavioural abnormalities, including autism (Campbell *et al*. 2008; Engel *et al*. 2008; Fernandez *et al*. 2010; Pucilowska *et al*. 2012). In addition, under certain circumstances ERK1 attenuates the ERK2 signal; indeed, ERK2 up‐regulation is seen in ERK1^KO^ mice (Selcher *et al*. 2003; Lefloch *et al*. 2008; Samuels *et al*. 2008) which, as shown herein, promotes neuronal death following HI. Despite these studies, to the best of our knowledge there is little information as to how suppression of ERK1 exacerbates injury in the neonatal HI mouse and further work is required to elucidate the role of ERK1 in injury response.

Global deletion of ERK1 and astrocyte‐specific ERK2 deletion resulted in a greater deleterious effect than global ERK1 deletion alone, with significantly higher expression of each damage marker. *In vitro* studies suggest a protective role of astrocytes to ODC precursor cells (Arai & Lo, 2010). As such they are suggested to be partially responsible for subsequent white matter injury, via H_2_O_2_ insult, by the up‐regulation of pERK. Application of U0126 abolishes the protective nature of astrocytes (Arai & Lo, 2010). Other *in vivo* studies looked at ERK2 excision under the GFAP promoter in the developing mouse and observed that whilst ODC precursor cells develop normally, there is a significant delay in maturation and reduced myelin production. (Fyffe‐Maricich *et al*. 2011). This suggests a developmental requirement of astrocytic ERK2 expression for normal ODC genesis.

Following oxidative stress, reactive oxygen species activation of ERK results in dysfunction of the mitochondrial outer membrane inducing cytochrome *c* release and cleavage of caspases 3 and 8 (Nowak, 2002; Nowak *et al*. 2006; Martin & Pognonec, 2010). ERK can further promote cytochrome *c* release via the up‐regulation of the pro‐apoptotic proteins Bax, PUMA and Bad (Cagnol & Chambard, 2010). Our study elucidates that ERK can be both protective and detrimental in neonatal HI injury depending on the cellular localisation of ERK.

Our data suggest that ERK2 deletion on its own seems to have ERK1 independent and cell‐specific function with a detrimental effect in neurons and a protective one in astrocytes. Our previous data demonstrate that inhibition of phosphorylated STAT3 (pSTAT3) has a neuroprotective effect in neonatal HI brain damage in a cell‐ and time‐specific manner (Hristova *et al*. 2016). While the pSTAT3 Tyr705 phosphorylation site, which is Jak2‐dependent, is associated with the transcriptional properties of STAT3, the Ser727 site (downstream of MAPK and ERK1/2) is responsible for recruitment to mitochondria and regulates functions alternative to transcription (Yang & Rincon, 2016). This suggests that inhibition of both ERK1 and 2 could result in mitochondrial dysfunction and might be a possible explanation for the detrimental effects observed when both ERK1 and ERK2 deletions combined were used. Although possible, this mechanism would require further investigation.

In neonatal brain injury studies, systemic injection of LPS leads to an up‐regulation of pro‐inflammatory cytokines and consequently increased neuronal and astroglial cell death (Jã *et al*. 2013). The synergistic nature of LPS to HI shows the same underlying white matter and grey matter lesion formation as those seen in human babies subject to infection as well as hypoxic ischemic encephalopathy (Wang *et al*. 2009; Kendall *et al*. 2011). LPS/TLR4/MyD88 induction of genes encoding pro‐inflammatory cytokines is mediated by the phosphorylation and nuclear translocation of ERK1/2 (An *et al*. 2002; Watts *et al*. 2011). MyD88 can form a functional complex directly with ERK1/2 via the recruitment of a scaffold protein MKP3 (tpl2) which prevents ERK dephosphorylation, rendering it constitutively active (Bandow *et al*. 2012). MKP3 had been previously implicated in the dysregulation of TLR2/MyD88 activation of ERK, resulting in transcription of its nuclear target Elk‐1.

Our data indicate that both single neuronal ERK2 mutation and double ERK1 and neuronal ERK2 mutation result in strong reduction in damage. Single mutants exhibited up to a 90% decrease in microglia and 40% decrease in astrocyte activation that correlate with a reduction in tissue loss and TUNEL+ cell death. Preservation of regional cell loss was validated by measurements of infarct. No reduction of cell death or tissue loss was observed with double mutation of ERK1 and neuronal ERK2.

*In vitro* dendritic cell cultures from ERK1 null mice show an increased expression of interleukin (IL)‐12p70 and a decrease of anti‐inflammatory IL‐10 secretion in response to TLR stimulation (Bandow *et al*. 2012). Hippocampal cultures exposed to combined LPS and interferon γ (IFNγ), a pro‐death cytokine, were susceptible to damage due to NO production by co‐cultured microglia (Xiao *et al*. 1996). Using the MEK inhibitor PD98059, IFNγ‐induced NO production was reduced by 40% (Bandow *et al*. 2012). In human monocytes, PD98059 reduced LPS induction of TNFα gene expression in a dose‐dependent manner. Indeed, inhibition of ERK decreased the release of several pro‐inflammatory cytokines including IL‐1 and IL‐18 (An *et al*. 2002; Gorina *et al*. 2011). To date, this is the first time evidence has been provided to implicate the capacity of ERK to modulate both neuronal and glial damage response following endotoxin‐sensitised ischaemia in the neonate.

Our results would need to be further confirmed in large animals as the rodent Rice–Vannucci model does not mimic the human condition optimally. This includes the level of white matter development, as well as the type of injury caused by the insult. In the rodent it is mostly severe injury with multiple infarctions involving both white matter and grey matter compared to diffuse apoptotic and relatively small necrotic areas in the human infant brain, affecting mostly white matter in most cases of periventricular leukomalacia resulting in cerebral palsy (Rumajogee *et al*. 2016).

As a conclusion our data confirm that neuronal ERK2 has a pivotal role in the development of neonatal HI brain damage. In contrast ERK1 and astrocytic ERK2 show a clear involvement in the pro‐survival response to insult. There is clear therapeutic potential for ERK2 inhibition after HI with the possibility of combined therapy to contest neurodegeneration by severe insult. Similar patterns of expression suggest that the therapeutic window of 1 h where inhibition could be beneficial is restricted and that pre‐emptive treatment during the developmental stage of infancy could diminish endogenous biochemical survival mechanisms, significantly worsening the outcome.

## Additional information

### Competing interests

None declared.

### Author contributions

L.T.: acquisition, analysis and interpretation of data; drafting and critically analysing the work. E.R.‐F.: acquisition and interpretation of data; drafting the work or revising it critically for important intellectual content. D.P.: conception of the work and revision of content; G.R.: conception of the work and revision of content; M.H.: acquisition, analysis and interpretation of data and revising the work. We confirm that all authors approved the final version of the manuscript and agree to be accountable for all aspects of the work in ensuring that questions related to the accuracy or integrity of any part of the work are appropriately investigated and resolved. All persons designated as authors qualify for authorship, and all those who qualify for authorship are listed. Work was carried out at the Institute for Womens Health, University, College London.

### Funding

This work was funded by SPARKS 07UCL02, and Welcome Trust grants: WT088646MA and WT089624MA.
